# Traditional Chinese medicine: An important source for discovering candidate agents against hepatic fibrosis

**DOI:** 10.3389/fphar.2022.962525

**Published:** 2022-08-23

**Authors:** Wen-Qing Li, Wen-Hao Liu, Die Qian, Jia Liu, Shi-Qiong Zhou, Lei Zhang, Wei Peng, Li Su, Hong Zhang

**Affiliations:** ^1^ School of Pharmacy, Chengdu University of Traditional Chinese Medicine, Chengdu, China; ^2^ Institute of Interdisciplinary Integrative Medicine Research, Shanghai University of Traditional Chinese Medicine, Shanghai, China; ^3^ Department of Pharmacy, Tenth People’s Hospital of Tongji University, Shanghai, China; ^4^ Hospital of Nursing, The Second Affiliated Hospital of Guilin Medical University, Guilin, China; ^5^ Department of Vascular Surgery, Yueyang Hospital of Integrated Traditional Chinese and Western Medicine, Shanghai University of Traditional Chinese Medicine, Shanghai, China; ^6^ Institute of Translational Medicine, Shanghai University, Shanghai, China

**Keywords:** natural products, hepatic fibrosis, hepatic stellate cells, extracellular matrix, hepatoprotection, mechanism, traditional Chinese medicines, Fufang-Biejia-Ruangan pill

## Abstract

Hepatic fibrosis (HF) refers to the pathophysiological process of connective tissue dysplasia in the liver caused by various pathogenic factors. Nowadays, HF is becoming a severe threat to the health of human being. However, the drugs available for treating HF are limited. Currently, increasing natural agents derived from traditional Chinese medicines (TCMs) have been found to be beneficial for HF. A systemic literature search was conducted from PubMed, GeenMedical, Sci-Hub, CNKI, Google Scholar and Baidu Scholar, with the keywords of “traditional Chinese medicine,” “herbal medicine,” “natural agents,” “liver diseases,” and “hepatic fibrosis.” So far, more than 76 natural monomers have been isolated and identified from the TCMs with inhibitory effect on HF, including alkaloids, flavones, quinones, terpenoids, saponins, phenylpropanoids, and polysaccharides, etc. The anti-hepatic fibrosis effects of these compounds include hepatoprotection, inhibition of hepatic stellate cells (HSC) activation, regulation of extracellular matrix (ECM) synthesis & secretion, regulation of autophagy, and antioxidant & anti-inflammation, etc. Natural compounds and extracts from TCMs are promising agents for the prevention and treatment of HF, and this review would be of great significance to development of novel drugs for treating HF.

## Introduction

**FIGURE 1:**
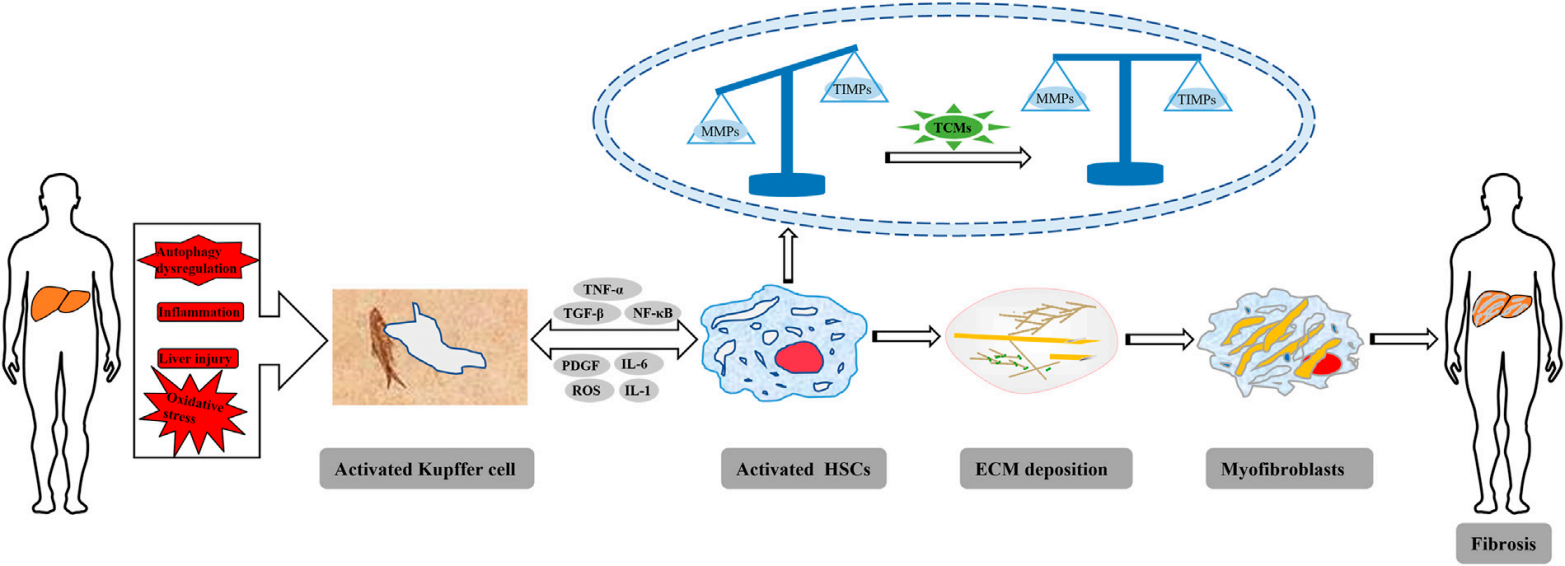
Schematic representation of the pathogenesis of HF.

**FIGURE 2:**
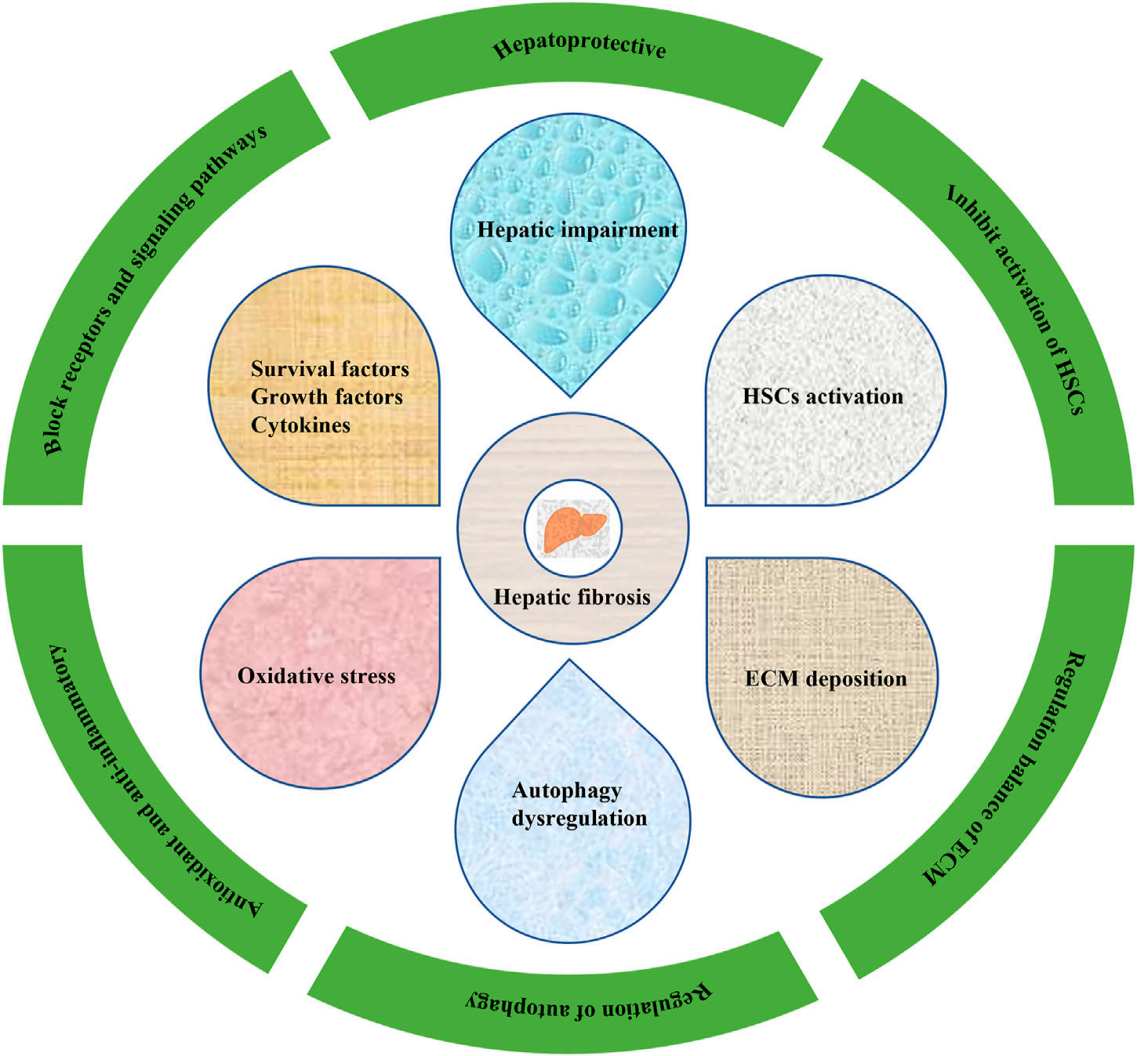
Pharmacological effects of TCMs on HF.

Hepatic fibrosis (HF) refers to abnormal hyperplasia of connective tissue in the liver caused by various pathogenic factors, and is a pathophysiological process of the repair of chronic liver injury. HF is a key step in the development of various chronic liver diseases to cirrhosis (Zhang C. Y. et al., 2016; Parola and Pinzani, 2019), and activation of HSCs is the central link in the development of HF, which is characterized by excessive and abnormal deposition of ECM in the liver (Chen S. R. et al., 2015; Povero et al., 2015) (Figure 1). In fact, HF is reversible (Roeb, 2018), and liver injury commonly has a process of HF in the process of liver repair and healing. However, if the injury factors cannot be removed for a long time, HF will develop into irreversible cirrhosis or even liver cancer (Tao et al., 2020). Liver cancer is one of the most commonly ones with high morbidity and mortality, and the epidemic investigations also reported that liver cancer is one of the currently leading causes of cancer-related death in the world (Mehta et al., 2015; Xiao et al., 2017). According to statistical analyses, approximately one million deaths annually were caused by liver cancer (Rawat et al., 2018; Martínez-Chantar et al., 2020). Therefore, it is important to treat HF before it evolves into cirrhosis or even liver cancer. However, nowadays, no specific therapeutic drugs could be used in clinical for treating HF, and western drugs don’t have good curative effects on HF but often result in some adverse reactions (Daniyal et al., 2019; Shan et al., 2019; Ma et al., 2020). Natural products have a general health promoting effect on the prevention and treatment of different diseases, especially liver ones. Polydatin, an active monomer from the *Poygonum cuspidatum* Sieb.et Zucc. (Polygonaceae), can protect hepatocytes from oxidative injury and ameliorate liver damage induced by carbon tetrachloride in rats (Peng et al., 2013). Hawthorn herbal preparation from *Crataegus oxyacantha* (Rosaceae) attenuates carbon tetrachloride-induced hepatic fibrosis *in vivo* via modulating oxidative stress and inflammation (Hamza et al., 2020). TCMs are derived from animals, plants and mineral agents with certain pharmacological activities, and herbal medicines and their formulas are dominant (Shan et al., 2019). TCMs have attracted much attention due to their unique advantages, such as wide sources, lower prices, few side effects, etc. Previous evidence suggests that many agents derived from TCMs could be beneficial for the treatment or prevention of HF (Shan et al., 2019). So far, more than 76 natural compounds with anti-hepatic fibrosis activities have been isolated and identified from TCMs, including alkaloids, flavones, quinones, terpenoids, saponins, phenylpropanoids, and polysaccharides, etc. The anti-hepatic fibrosis effects of these compounds include hepatoprotection, inhibition of hepatic stellate cells (HSC) activation, regulation of extracellular matrix (ECM) synthesis & secretion, regulation of autophagy, and antioxidant & anti-inflammation, etc. (Figure 2). In the present review, we summarized the potential anti-hepatic fibrosis components from TCMs and their possible molecular mechanisms, which would be of great significance to develop novel drugs against hepatic fibrosis.

### Pathogenesis of hepatic fibrosis

#### HSCs-mediated hepatic fibrosis

Hepatic stellate cells (HSCs) are a kind of non-parenchymal cells of liver, located in the disse space, accounting for 5–10% of total of hepatocytes, and maintain close interaction with hepatic sinusoidal endothelial cells and hepatocytes (Puche et al., 2013; de Oliveira da Silva et al., 2017). HSCs are the most important source cells of extracellular matrix (ECM) in HF, and activation of HSCs is a key and central step in the development of HF (Cai et al., 2020). In normal liver, HSCs are in a quiescent state without α-smooth muscle actin (α-SMA) secretion, have low proliferative activity and low ability to synthesize collagen, and their main functions are to store and metabolize VitA lipid droplets. When the liver is damaged by inflammation or mechanical stimulation, HSCs could be activated, and their phenotype changes from “quiescent” to “active” (Dewidar et al., 2019).

HSC activation includes two main stages: initiation phase and persistence phase. The priming stage refers to the changes in gene expression in early stage and in cell phenotype caused by stimulating factors such as cytokines. When liver parenchymal cells are injured, cytokines are created by the neighboring hepatocytes, Kupffer cells through paracrine action, such as tumor necrosis factor α (TNF-α), transforming growth factor β (TGF-β), insulin growth factor (IGF), hepatocyte growth factor (HGF), and platelet-derived growth factor (PDGF), etc. (Tsuchida and Friedman, 2017), which can significantly promote the activation, division, proliferation, transformation of HSCs into myofibroblasts (MFBs) to synthesize ECM. Furthermore, activated HSCs can also secrete cytokines such as TNF, TGF and PDGF to sustain the activation, leading to further aggravation of HF. Persistent phase refers to the maintenance of HSC activation due to the role of above factors, increasing the deposition of ECM, resulting in HF. Thus, HSC activation is regulated by both autocrine and paracrine at this stage (Chen et al., 2008; Wallace et al., 2015; de Oliveira da Silva et al., 2017; Tsuchida and Friedman, 2017; Barcena-Varela et al., 2019). Obviously, activation of HSC is the cytological basis of HF.

#### ECM-mediated hepatic fibrosis

Extracellular matrix (ECM) is mainly composed of collagen, non-collagen glycoproteins (including fibronectin, laminin, etc.), elastin, proteoglycans and aminoglycans, whose intercellular adhesion structure is interstitial connective tissue. The formation of HF is due to the deposition of large amounts of ECM in the liver, and HSC activation is the main cause of ECM production (Seki and Brenner, 2015; Zhao et al., 2019). In normal liver, the synthesis and degradation of ECM mainly composed of collagens IV and VI maintain a dynamic balance, which regulates cell growth and metabolic activities. When the liver is damaged, the synthesis of ECM is greater than degradation. ECM proliferation leads to a large amount of collagen deposition. The original collagens IV and VI are replaced by collagen I, III and fibronectin to promote the formation of fibrosis. On the other hand, ECM stimulates HSC activation and proliferation. Activated HSCs can produce matrix metalloproteinases (MMP) -9 and -13, breaking the balance mechanism between MMPs and tissue inhibitors of metalloproteinases (TIMPs), which leads to partial hydrolysis of ECM deposited in large amount, reducing the occurrence of fibrosis (Nunes de Carvalho et al., 2013; Motawi et al., 2014; Karsdal et al., 2015). MMPs are the main enzymes degrading ECM and can specifically hydrolyze collagen and other matrix proteins, while TIMPs can prevent ECM degradation and promote the formation of fibrosis by inhibiting the activity of MMPs (Pinlaor et al., 2010; Baig et al., 2016). Therefore, regulating the balance of MMPs/TIMPs is essential for the degradation of ECM.

#### Autophagy-mediated hepatic fibrosis

Autophagy is a process of degrading excess or damaged organelles or other components in the cell with the participation of lysosomes, is essential for cell survival, differentiation, growth, and homeostasis (Tao et al., 2020). Autophagy not only regulates hepatocyte functions but also impacts on non-parenchymal cells, such as endothelial cells, macrophages and hepatic stellate cells (Allaire et al., 2019). Dysregulation of autophagy has been implicated in many liver diseases, and its modulation has also been suggested as a potential new therapeutic strategy for hepatic fibrosis. Studies have shown that autophagy has important regulatory effects on hepatic stellate cells (HSCs) energy metabolism, and then affects the activation state of HSCs (Hou et al., 2022), and further affects the progression of hepatic fibrosis. It is reported that, on the one hand, autophagy has a direct promoting effect on liver fibrosis because it could participate in the digestion of lipid droplets and provide energy for the activation of HSCs; on the other hand, autophagy could inhibit the emergence of hepatic fibrosis through anti-inflammatory effects (Sun et al., 2021). Undoubtedly, autophagy is seen in both physiological and pathological processes of the organism, and whether the role it plays is positive or negative has not been fully elucidated, and research on hepatic fibrosis, in particular, is of concern.

#### Oxidative stress-mediated hepatic fibrosis

Oxidative stress refers to a state in which reactive oxygen species (ROS) are produced and/or the antioxidant defense function is weakened in the body or cells during the transition, resulting in a serious imbalance between the two and tissue and cell damage (van der Pol et al., 2019). When the liver is injured, firstly, ROS production increases and clearance decreases in the liver tissue, Kupffer cells are activated to release many inflammatory mediators and cytokines (Gong and Yang, 2020). On the other hand, chemokines secreted by Kupffer cells recruit other immune cells to the site of injury to aggravate inflammatory injury, which can directly stimulate HSCs activation and proliferation (Ghatak et al., 2011) through autocrine and paracrine, resulting in a large deposition of ECM, thereby promoting the formation of hepatic fibrosis (Yang J. J. et al., 2013; Richter et al., 2015).

### Hepatic fibrosis mediated by cytokines and signaling pathways

#### TGF-β signaling pathway

TGF-β, an activator of MFBs, is a central regulator of chronic liver disease and potent profibrotic cytokine (Deng et al., 2012; Fabregat et al., 2016). When the liver is damaged, Kupffer cells produce TGF-β in a paracrine manner, causing HSC activation. Activated HSCs can also secrete a large amount of TGF-β, which continues to stimulate the active expression of TGF-β, forming a step-by-step amplification effect (Dooley and ten Dijke, 2012). When activated TGF-β binds to the membrane receptor in HSC, TGF-β receptor activates and phosphorylates R-smad protein that binds to this receptor. Activated R-smad forms heteropolymers with co-Smad protein shared by the cytoplasm and translocated into the nucleus. Smad protein regulates transcription through target gene, thereby triggering the overexpression of fibrotic genes. Many studies have shown that TGF-β1/Smad-2 and Smad-3 are major downstream regulators promoting TGF-β-mediated HF, while Smad-7 is a negative feedback regulator of the TGF-β1 signaling pathway which has a protective effect on TGF-β-mediated HF (Xu et al., 2016; Hu H. H. et al., 2018; Dewidar et al., 2019).

#### NF-κB signaling pathway

NF-κB is an important nuclear transcription factor in cells (Robinson and Mann, 2010), and a major regulator of inflammatory response and apoptosis (Sun and Karin, 2008). In HF, NF-κB can be activated by LPS, TNF and IL-1β, leading to enhanced TGF-β signaling and HSC activation, while NF-κB secretion of macrophage recruitment chemokines and its anti-apoptotic effect can promote the formation of fibrosis (Elsharkawy and Mann, 2007; Tacke et al., 2009). On the other hand, NF-κB plays an anti-fibrotic role by inhibiting the transcription of COL1a1 gene (Zhang Y. Z. et al., 2019). Therefore, NF-κB may act as both pro-fibrotic and anti-fibrotic effects.

#### TLR4 signaling pathway

TLR4 is an important regulator of hepatic inflammatory response in chronic injury (Roy et al., 2016). In the liver, HSC has a complete TLR4 signaling pathway. TLR4 can be activated by LPS and HMGB1, and the activated TLR4 could induce downregulation of TGF-β pseudo receptor BAMBI through the downstream NF-κB signaling pathway, leading to increased activity of TGF-β1 (Roh and Seki, 2013; Li Z. et al., 2019; Nakanishi et al., 2019; Dong Z. et al., 2020). Meanwhile, TLR4 activation in HSCs promotes the recruitment of Kupffer cells, which in turn promotes the release of TGF-β, leading to the activation of HSCs and the formation of fibrosis (Pradere et al., 2010; Gao et al., 2011).

#### Wnt/β-catenin signaling pathway

The Wnt signaling pathway is a signal transduction one that activates downstream channels by binding of ligand protein Wnt and membrane protein receptors, enabling extracellular signals to be transmitted into cells (Thompson and Monga, 2007). In the classical Wnt signaling pathway, its membrane receptor is β-catenin, so it is also called Wnt/β-catenin signaling pathway (Huang et al., 2018). The canonical Wnt signaling pathway is active in HSCs, which promotes the formation of fibrosis by inducing the activation and survival of HSCs, leading to increased expression of type I collagen and α-SMA (Wang J. N. et al., 2018; Nishikawa et al., 2018; Li L. Y. et al., 2019; Rong et al., 2019).

#### PDGF signaling pathway

Platelet-derived growth factor (PDGF) is the most effective one to stimulate HSC proliferation, differentiation, and migration (Ying et al., 2017). PDGF also promotes collagen production and deposition, and converts HSCs into MFBs. The receptor of PDGF (PDGFR) belongs to the protein tyrosine kinase receptor family and has the function of protein tyrosine kinase, mainly located in vascular endothelial cells, fibroblasts and Kupffer cells (Borkham-Kamphorst and Weiskirchen, 2016). When liver injury occurs, hepatocytes release excessive PDGF, which binds to and activates PDGFR to initiate a variety of protein cascade phosphorylation signal transduction pathways, thereby causing a cellular response. The main downstream signaling pathways of PDGF include: 1) Ras/ERK/MAPK pathway; 2) AKT/mTOR/p27 pathway; 3) JAK/STAT pathway; 4) Ca^2+^ pathway (Kocabayoglu et al., 2015; Borkham-Kamphorst and Weiskirchen, 2016; Ying et al., 2017). Collectively, PDGF continuously activates and proliferates through autocrine and paracrine mechanisms, which leads to mitosis of HSCs and excessive deposition of ECM, promoting the formation of fibrosis.

## Effects of extracts/monomers from TCMs against hepatic fibrosis

### Hepatoprotective

Liver injury can be caused by a variety of reasons, including alcohol, drug poisoning, autoimmune overreaction, etc. In the process of liver injury, activated HSC could produce excessive ECM and promote the formation of fibrosis (Hu et al., 2020a). Therefore, search for effective hepatoprotective agents has attracted extensive attention (Li et al., 2020; Wu et al., 2020). It has been reported that some monomers extracted from TCMs have hepatoprotective effects, such as glycyrrhetinic acid, curcumin, emodin, baicalin and some plant polysaccharides (Stanković et al., 2017), which can reduce the occurrence of fibrosis by protecting the liver. Chen et al. (Chen et al., 2013) found that glycyrrhetinic acid (25, 50, 100 mg/kg) might enhance the expression of its target genes and the activity of antioxidant enzymes by activating the nuclear translocation of Nrf2 to protect the liver of mice from oxidative stress. It can be used as a feasible candidate drug to prevent HF. Kong et al. (Kong et al., 2020) found that curcumin (100, 200, 400 mg/kg) could blunt the epithelial-mesenchymal transition (EMT) of hepatocytes to reduce the occurrence of HF.

### Inhibiting activation and promoting apoptosis in HSC

Activation of HSCs is a major event in the pathogenesis of HF (Jin et al., 2016), and inhibiting activation and proliferation of HSC is a possible step in the intervention of HF. Following observing the effect of conophylline (0.9 μg/g) on the expression of α-SMA and collagen I in rat HSCs. Kubo et al. (2014) found that conophylline reduced the expression of α-SMA and collagen-1, thereby inhibiting HSC activation. Ligustrazine (50, 100, 200 mg/kg) inhibited the production, migration, metabolism, and contraction of angiogenic factors in HSCs (Zhang et al., 2018). Artesunate (50, 100, 200 mg/kg) significantly induced apoptosis of activated HSCs in CCl_4_-induced mouse hepatic fibrosis (Kong et al., 2019). Consistent with the experimental results *in vivo*, artesunate treatment markedly repressed HSC activity, raised cell mortality, increased lipid peroxides and reduced antioxidant capacity *in vitro*.

### Regulation synthesis, secretion and degradation of ECM

Overaccumulation of ECM is the initial stage of HF, and HSC activation is a central event in the pathogenesis of HF (Xie et al., 2021). Therefore, regulation of the synthesis and degradation of ECM is of great significance for the treatment of HF. Oroxylin A (20, 30, 40 mg/kg) could significantly inhibit the deposition of ECM in CCl_4_-induced mouse HF model, suggesting the possibility of oroxylin A for the treatment of HF (Chen et al., 2018). In the experiment of CCl_4_-induced hepatic fibrosis in rats, tanshinone IIA (2.5, 5.0, 10 mg/kg) significantly improved liver function, reduced liver injury, decreased ECM accumulation, inhibited HSC proliferation and activation, thus improving fibrosis (Shi et al., 2020).

### Regulation of autophagy

Numerous studies suggest a key role of autophagy dysbiosis in the progression of hepatic fibrosis. MFBs are considered to be the main products of HSCs, and their massive production and activation are key to the development of hepatic fibrosis (Kisseleva and Brenner, 2021). More recently, in a model of hepatic fibrosis in rats injected intraperitoneally with CCl_4_, Kong et al. recently provided evidence that curcumin (100, 200, 400 mg/kg) inhibited TGF-β/Smad signaling transmission by activating autophagy, thereby inhibiting EMT, further inhibiting the production of MFBs to protect against hepatic fibrosis (Kong et al., 2020). In addition, *in vitro*, curcumin (10, 20, 30 μM/L) suppressed levels of ROS and oxidative stress in hepatocytes, and modulated upstream signaling pathways of autophagy AMPK and PI3K/AKT/mTOR, leading to an increase of the autophagic flow in hepatocytes (Kong et al., 2020). Furthermore, studies have also revealed the role of autophagy in CCl_4_-induced hepatic fibrosis, and to further examine the molecular mechanisms after treatment oroxylin A, at the same time leading to a decrease in the levels of gene expression of the main actors of liver damage, such as ALP, AST, ALT, TGF-β1, TNF-α, α-SMA, and the expression of autophagy makers, such as LC3-B, Atg3, Atg4, Atg5, Atg6, Atg7, Atg9, Atg12, Atg14 Beclin1, and p62, etc. was significantly up-regulated, consequently, oroxylin A is required to activate autophagy to attenuate hepatic fibrosis and HSCs activation (Chen et al., 2018). Hence, based on these studies, we reasonably point to the possibility of using natural drugs to treat hepatic fibrosis by regulating autophagy.

### Antioxidant and anti-inflammatory effects

Oxidative stress can create a vicious circle of fibrous deposition, and persistent inflammation is an initiation and maintenance factor of HF (Ruart et al., 2019; Bai et al., 2020). Therefore, antioxidation and inhibition of inflammation can effectively improve HF. The natural compouds exerting antifibrosis effect through antioxidation and anti-inflammation mainly include *acanthopanax senticosus* alkaloid A, berberine, betaine, astragaloside IV, breviscapine, rhein, oxymatrine, cucurbitacin B, and so on. For example, berberine (50 mg/kg) has antioxidant and anti-inflammatory activities and is a potential drug for the treatment of HF (Eissa et al., 2018). Astragaloside IV (50 mg/kg) inhibited the activation of HSC by inhibiting oxidative stress and p38 MAPK pathway, thus achieving the anti-hepatic fibrosis effect (Li et al., 2013). Breviscapine (15, 30 mg/kg) attenuated CCl_4_-induced liver injury in mice by inhibiting inflammatory apoptotic response and ROS production (Liu Y. et al., 2018). In addition, nonalcoholic steatohepatitis (NASH) is characterized by histological lobular inflammation and hepatocyte ballooning, the active form of non-alcoholic fatty liver disease NAFLD, and is associated with disease progression, development of fibrosis, eventually, cirrhosis and hepatocellular carcinoma (Castera et al., 2019). A mouse model of NASH was established by feeding a high-fat/high-cholesterol diet, for 16 weeks, and breviscapine (15 and 30 mg/kg) was administered daily by oral gavage after 8 weeks of treatment, significantly reduced lipid accumulation, inflammatory cell infiltration, liver injury, and fibrosis in mice. Further RNA-sequencing and multiomics analyses indicated that breviscapine inhibited TGF-β-activated kinase 1 (TAK1) -dependent signaling to alleviate NASH (Lan et al., 2022). Moreover, *in vitro* and *in vivo* results of the experiment showed that gastrodin (25, 50, 100, 200 μg/ml; 10, 20, 50 mg/kg) activated the AMPK/Nrf2 pathway, ameliorated hepatic oxidative stress/proinflammatory response and significantly improved metabolic disorders in NAFLD (Qu et al., 2016). These findings will provide scientific basis for the clinical application of natural botanical compounds to treat HF in the future.

### Blockade of receptors and signaling pathways

TGF-β, PDGF and PPARγ are important fibrogenic factors, and blocking the binding of these cytokines to HSC membrane receptors or inactivating their signal transduction pathways can all achieve anti-fibrotic effects (Zhang et al., 2013; Borkham-Kamphorst and Weiskirchen, 2016; Frangogiannis, 2020; Sharma et al., 2020). For example, caffeine (37.5 mg/kg) inhibited the formation of HF by inhibiting the expression of TGF-β-stimulated connective tissue growth factor (CTGF) in hepatocytes through PPARγ and Smad2/3 pathways (Gressner et al., 2008). A rat model of liver injury was established and treated with different doses of baicalin (25, 50, 100 mg/kg). The results showed that baicalin reversed the biochemical indicators of liver injury, inhibited the upregulation of TGF-β1 expression, and enhanced the expression of PPARγ (Qiao et al., 2011).

## Traditional applications of TCMs against hepatic fibrosis

In traditional Chinese medicine system, the HF belongs to the scopes of “hypochondriac pain,” “jaundice,” “liver impediment” and “tympanites” and commonly induced by “deficiency of Qi and blood stasis,” “liver Qi stagnation,” “moist-heat collecting in the splenetic and stomach systems” and “damp abundance due to splenic asthenia,” etc. (Zhang X. Y. et al., 2019; Xu, 2020). In China, TCMs have been comprehensively used for treating HF for thousands of years based on the functions of “promoting blood circulation and removing blood stasis,” “tonifying Qi,” “soothing liver and strengthening spleen” and “clearing away heat and toxic material,” etc. (Duan et al., 2020; Xu, 2020). The commonly used TCMs include the *Danshen* (roots of *Salvia miltiorrhiza* Bge*.*, Lamiaceae)*, Gancao* (roots of *Glycyrrhiza uralensis* Fisch., Leguminosae)*, Banxia* (roots of *Pinellia ternata* (Thunb.) Breit., Araceae)*, Renshen* (roots of *Panax ginseng* C.A.Mey., Araliaceae)*, Huangqin* (roots of *Scutellaria baicalensis* Georgi, Lamiaceae)*, Shengjiang* (roots of *Zingiber* officinale Rosc., Zingiberaceae), *Baizhu* (roots of *Atractylodes macrocephala* Koidz., Asteraceae), and *Yujin* (roots of *Curcuma wenyujin* Y.H. Chen et C. Ling, Zingiberaceae), etc. (Duan and Wang., 2020). TCMs are often applied in clinical to treat diseases in the form of formula, and importantly, TCM formula is also recognized as one of promising therapies against HF due to its feature of “multiple components, multiple targets and multiple pathways” (Li, 2020; Sun, 2020).

The Fufang-Biejia-Ruangan pill (FBRP) is the first clinically approved classic Chinese herbal formula against fibrosis in China (Zhang et al., 2020). In CCl_4_-induced hepatic fibrosis rat model, daily orally administration with FBRP, at doses of 0.55 g/kg, for 8 weeks, decreased the serum levels of ALT, AST, PCIII, HA, LN and IV-C, alleviated the level of infiltration of leukocytes, necrosis, bile duct proliferationa, and collagen deposition as well as downregulated reduced the protein expression of TGF-β1 and Smad3 in the rat liver (Yang F. R. et al., 2013). Further *in vitro* investigations demonstrated that FBRP significantly suppressed HSC-LX-2 cell proliferation and reduced hydroxyproline content (Yang F. R. et al., 2013). These results indicated that FBRP shows significant anti-hepatic fibrosis effects *via* inhibition of TGF-β/Smad signaling pathway may be an underlying mechanism. Moreover, *in vivo* model of diethylnitrosamine (DEN) induced hepatocellular carcinoma (HCC) in rats, daily orally administrated with FBRP at doses of 1 g/kg, for 18 weeks, effectively reduced the serum levels of ALT, AST, ALP, TP, HA and alpha fetoprotein (AFP), significantly suppressed the liver tissues expression of p-PI3K, p-AKT, p-IKkB, and p-NF-κB, as well as the ratios of p-IKkB/total IKkB and p-NF-κB/total NF-κB, suggested FBRP may be a promising candidate drug for reduction of fibrogenesis and prevention of HCC *via* blocking PI3K/AKT/NF-κB activation (Zhang et al., 2020). Meanwhile, *in vitro*, Huh7 cells were treated with 5.0 and 10.0 mg/ml FBRP for 24 h, suppressed HCC cell proliferation and induced cell cycle arrest, obviously downregulated the expression of related factors in the PI3K/AKT/NF-κB signaling pathway (Zhang et al., 2020). Notably, network-based pharmacological strategies, Zhang et al. (2020) also provided a reasonable data for unveiling that FBRP may block PI3K/AKT/NF-κB activation, further highlighting the multi-target anti-HCC. Similarly, *in vivo*, orally administrated with FBRP, at doses of 0.625 g/kg, once a day during the 7 weeks, detection of biochemical indicators, fibrosis-related index and the MAPK pathway involved factors at the end of experiment. The results showed that FBRP dramatically the serum level of ALT, AST, HA, LN, IV-C and PCIII, significantly reduced the levels of α-SMA, TNF-α, IL-13, p-p38, p-ERK both in serum and liver tissues in rats (Cai et al., 2010).

Besides, *in vivo* model of CCl_4_-induced hepatic fibrosis in rats, daily orally administration with FZHY formula, at doses of 4.6 g/kg, for 9 weeks, alleviated the hepatic lobular distortion and collagen fiber accumulation, improved the hepatocellular steatosis and ballooning. Moreover, FZHY dramatically elevated the expression of Hexokinase 2 (Hk2) and glutamine synthetase (Gs), regulated the both glucose metabolism and amino acid metabolism as well as reduced dehydrogenase 1 (Adh1), acetyl-CoA synthetase 2 (Acss2) and glutamic-pyruvictransaminase (Gpt) (Hu et al., 2019), suggested FZHY has good anti-hepatic fibrosis effect via altering the metabolic pathways. Fu et al. (2021) found a novel Chinese medicine, namely JY5 formula, composed of salvianolic acid B, schisantherin A and amygdalin, the main active ingredients of FZHY, and evaluated its anti-hepatic fibrosis activity both *in vitro* and *in vivo*. In the CCl_4_-and BDL-induced hepatic fibrosis rat experiment, after treatment with JY5 (salvianolic acid B: 16 mg/kg, schisantherin A: 2 mg/kg, amygdalin: 0.5 mg/kg), significantly alleviated hepatic injury and collagen deposition, the expressions of Jagged1, Notch2, Notch3, Notch4 and recombination signal binding protein-κB (RBP-κB) were significantly decreased. Meanwhile, consistent results were obtained in CCl_4_-induced hepatic fibrosis in mice, after JY5 (salvianolic acid B, 16 mg/kg amygdalin, 0.5 mg/kg schisantherin A, 2 mg/kg) treatment (Fu et al., 2021). These results suggest that JY5 could significantly inhibit the activation of Notch signaling pathway in CCl_4_-and BDL-induced liver fibrosis. Moreover, *in vitro* results showed, compared to the TGF-β1-induced activation of hepatic stellate cell line (LX-2) group, after treatment with JY5, at concentrations of 6.587, 13.174 and 26.348 μg/ml, the mRNA levels of α-SMA, Col-I, Jagged1, Notch2, Notch3 and RBP-кB were significantly reduced, the expression above these proteins were significantly reduced in the JY5-treated groups, and the high-dose JY5 group more significantly (Fu et al., 2021). The commonly clinically used TCM formulas against HF were displayed in Table 1.

**TABLE 1:** Traditional Chinese medicine formulas with anti-hepatic fibrosis effect.

TCM formula	Compositions	Mechanisms	Refs
Fufang-Biejia-Ruangan pill	Carapace of *Trionyx sinensis Wiegmann*; Roots of *Paeonia lactiflora* Pall.; *Cordyceps sinensis* (BerK.) Sacc.; Roots of *Panax notoginseng* (Burk.) F.H.Chen; *Hominis placenta*; Fruits of *Forsythia suspensa* (Thunb.) Vahl; Roots of *Angelica sinensis* (Oliv.) Diels; Roots of *Curcuma phaeocaulis* Val.; Roots of *Codonopsis pilosula* (Franch.) Nannf.; Roots of *Astragalus membranaceus* (Fisch.) Bge.; Roots of *Isatis indigotica* Fort.	Inhibiting activation of TGF-β/Smad signaling; Inhibiting activation of PI3K/AKT/NF-κB signaling; Downregulating MAPK pathway	Cai et al. (2010), Feng-Rui Yang et al. (2013), Zhang et al. (2020)
Fuzheng Huayu formula	Roots of *Salvia miltiorrhiza* Bge.; Seeds of *Prunus persica* (L.) Batsch; Whole herb of *Gynostemma pentaphyllum* (Thunb.) Makino; Fruits of *Schisandra chinensis* (Turcz.) Baill., *Cordyceps sinensis* (BerK.) Sacc.	Altering the metabolic pathways and regulating gene expression of involved metabolic enzymes; Inhibiting the Notch signaling pathway	Hu et al. (2019), Fu et al. (2021)
Yinchenhao decoction	Whole herb of *Artemisia capillaris* Thunb.; Fruits of *Gardenia jasminoides* Ellis; Roots of *Rheum palmatum* L.	Regulating bile acid metabolism and TGF-β/Smad/ERK signaling pathway	Cai et al. (2018)
PienTze Huang	Gallstone of *Bos taurus domesticus Gmelin Cow bezoar*; Secretion of *Moschus berezovskii Flerov*; Roots of *Panax notoginseng* (Burk.) F.H.Chen; Gallbladder of *Zaocys dhumnades Contor*	Suppressing NF-κB pathway and promoting HSC apoptosis; Enhancing the immune process	Zheng et al. (2019), Zhu et al. (2021)
Ganshuang granule	Roots of *Codonopsis pilosula* (Franch.)Nannf.; Roots of *Bupleurum chinense* DC.; Roots of *Paeonia lactiflora* Pall.; Roots of *Angelica sinensis* (Oliv.) Diels; Sclerotium of *Poria cocos* (Schw.) Wolf; Roots of *Atractylodes macrocephala* Koidz.; Whole herb of *Taraxacum mongolicum* Hand. Mazz.; Roots of *Polygonum cuspidatum Sieb.et Zucc.*; Ear of *Prunella vulgaris* L.; Roots of *Salvia miltiorrhiza* Bge.; Seeds of *Prunus persica* (L.) Batsch; Carapace of *Trionyx sinensis Wiegmann*; Immature fruit of *Citrus aurantium* L.	Inhibiting HSCs activation	Shi et al. (2017)
Herbal compound 861	Roots of *Salvia miltiorrhiza* Bge; Roots of *Astragalus membranaceus* (Fisch.) Bge.; Roots of *Bupleurum chinense* DC.; Caulis of *Spatholobus suberectus Dunn*; Roots of *Ligusticum chuanxiong* Hort.; Roots of *Cyperus rotundus* L.; Roots of *Paeonia lactiflora* Pall.; Peel of *Citrus reticulata Blanco*; Roots of *Angelica sinensis* (Oliv.) Diels; Flower of *Carthamus tinctorius* L.	Increasing SnoN protein expression and inhibiting the TGF-β1/Smad signaling pathway	Chi et al. (2018)
Xiaochaihu decoction	Roots of *Bupleurum chinense* DC.; Roots of *Scutellaria baicalensis* Georgi; Roots of *Codonopsis pilosula* (Franch.) Nannf.; Roots of *Pinellia ternata* (Thunb.) Breit.; Roots of *Glycyrrhiza uralensis* Fisch.; Roots of *Zingiber officinale* Rosc.; Fruits of *Ziziphus jujube* Mill.; Roots of *Panax ginseng* C.A.Mey.	Up-regulating Nrf2 pathway against oxidative stress and inhibiting activated HSCs	Li et al. (2017)
Liuwei wuling tablets	Fruits of *Schisandra chinensis* (Turcz.) Baill; Fruits of *Ligustrum lucidum* Ait.; Fruits of *Forsythia suspensa* (Thunb.) Vahl; Roots of *Curcuma wenyujin* Y.H. Chen et C. Ling; Roots of *Curcuma aeruginosa* Roxb.; *Ganoderma lucidum* (Leyss.ex Fr.) Karst.	Modulating TGF-β/Smad and NF-κB signaling pathways	Huimin Liu et al. (2018)
Anluo huaxian pill	Roots of *Rehmannia glutinosa* Libosch.; Roots of *Panax notoginseng* (Burk.) F.H.Chen; *Whitmania pigra* Whitman; *Pheretima aspergillum* (E.Perrier); *Bombyx mori Linnaeus*; Roots of *Atractylodes macrocephala* Koidz.; Roots of *Curcuma wenyujin* Y.H. Chen et C. Ling; Gallstone of *Bos taurus domesticus Gmelin Cow bezoar*; Shell of *Arca subcrenata Lischke;*Root and bark of *Paeonia suffruticosa* Andr.; Roots of *Rheum palmatum* L.; Fruits of *Hordeum vulgare* L.; Gizzard of *Gallus gallus domesticus Brisson*; Shell of *Bubalus bubalis Linnaeus*	Improving liver function, inhibiting the activation of hepatic stellate cells, enhancing the expression of MMP-13, and inhibiting the expression of MMP-2 and TIMP-1/2	Wang L. et al. (2019)
Huang Qi decoction	Roots of *Astragalus membranaceus* (Fisch.) Bge.; Roots of *Glycyrrhiza uralensis* Fisch.	Inhibiting Notch signaling activation	Zhang et al. (2017)
Qinggan Huoxue decoction	Roots of *Bupleurum chinense* DC.; Roots of *Scutellaria baicalensis* Georgi; Roots of *Salvia miltiorrhiza* Bge.; Carapace of *Trionyx sinensis* Wiegmann; Roots of *Pueraria lobata* (Willd.) Ohwi	Inhibiting TGF-β1/Smad1 signaling pathway	Wu et al. (2016)
Gan-fu-kang	Fruits of *Schisandra chinensis* (Turcz.) Baill; Roots of *Pseudostellaria heterophylla* (Miq.) Pax ex Pax et Hoffm.; Whole herb of *Hedyotis diffusa* Willd.	Downregulating Wnt/Ca^2+^ signaling	Jia et al. (2016)
Taohong Siwu decoction	Roots of *Rehmannia glutinosa* Libosch.; Roots of *Paeonia lactiflora* Pall.; Roots of *Angelica sinensis* (Oliv.) Diels; Roots of *Ligusticum chuanxiong* Hort.; Seeds of *Prunus persica* (L.) Batsch; Flos of *Carthamus tinctorius* L.	Reducing inflammatory reaction and VEGF expression and downstream signaling transduction	Xi et al. (2016)
Xia-yu-xue decoction	Roots of *Rheum palmatum* L.; Seeds of *Prunus persica* (L.) Batsch; *Eupolyphaga sinensis* Walker	Inhibiting hepatic stellate cell activation by targeting NF-κB and TGF-β1 signaling pathways	Liu et al. (2015)
Ger-Gen-Chyn-Lian decoction	Roots of *Pueraria lobata* (Willd.) Ohwi; Roots of *Scutellaria baicalensis* Georgi; Roots of *Coptis chinensis* Franch.; Roots of *Glycyrrhiza uralensis* Fisch.	Inhibiting hypoxia-inducible factor-1α-mediated pathway	Chang et al. (2019)
Dahuang zhechong pill	Roots of *Rheum palmatum* L.; Roots of *Scutellaria baicalensis Georgi*; Roots of *Glycyrrhiza uralensis* Fisch.; Seeds of *Prunus persica* (L.) Batsch; Seeds of *Prunus armeniaca* L.; Roots of *Paeonia lactiflora* Pall.; Roots of *Rehmannia glutinosa* Libosch.; *Whitmania pigra* Whitman; *Eupolyphaga sinensis* Walker; *Toxicodendri Resina*; *Tabanus bivittatus Matsumura*; *Holotrichia diomphalia Bates*	Decreasing the secretion of TNF-α and IL-13 through downregulating p38 and ERK phosphorylation	Cai et al. (2010)
Compound kushen injection	The roots of Kushen and Baituling with several identified bioactive alkaloids, including oxymatrine, matrine, oxysophocarpine, and sophocarpine	Rebalancing TGF-β/Smad7 signaling in HSCs	Yang et al. (2021)
Yiguanjian decoction	Radix rehmanniae (*Rehmannia glutinosa* (Gaetn.) Libosch. ex Fisch. et Mey.), Radix glehniae (*Glehnia littoralis* Fr. Schmidt ex Miq.), Radix ophiopogonis (*Ophiopogon japonicus* (Thunb.) Ker-Gawl.)*,* Fructus lycii (*Lycium barbarum* L.), Radix Angelicae sinensis (*Angelica sinensis* (Oliv.) Diels)*,* and Fructus toosendan (*Melia toosendan* Sieb. et Zucc.)	Inhibition of M1 polarization of macrophages	Xu et al. (2021)
Kangxian ruangan capsule	Artemisia capillaris (20 g), *Astragalus membranaceus* (10 g), Turtle shell (10 g); minister drug: Coix seed (20 g), *Salvia miltiorrhiza* (30 g), *Angelica sinensis* (10 g), *Curcuma zedoaria* (10 g), Ground beetle (10 g); assistant drug: *Panax notoginseng* (6 g), peach seed (10 g), Parched pangolin scales (6 g), ambassador drug: *Rhizoma atractylodis* macrocephalae (10 g)	Reducing HSC activation by suppressing the TGF-β1 and TLR4 signaling pathways	Liu et al. (2021)
Yinchen Wuling powder	artemisia Capillaris Herba, Polyporus Umbellatus, Alismatis Rhizoma, Atractylodes Macrocephalae Rhizoma stir-fried with wheat bran, Poria and Cinnamomi Ramulus	Regulating the imbalance of gut microbiota	Zhang et al. (2021)
Xueshisanjia no-decoction granule prescription	Turtle shell 15 g, pangolin 3 g, batryticated silkworm 10 g, ground beetle 10 g, peach kernel 10 g, bupleurum 5 g (total 53 g)	Promoting the miR-29b-3p/VEGFA axis	Zhang et al. (2022)
Si-Ni-San	Roots of *Bupleurum scorzonerifolium* Willd.; Roots of *Paeonia lactiflora* Pall.; *Citrus aurantium* L.; *Glycyrrhiza uralensis* Fisch.	Alleviating inflammation, ECM accumulation, aberrant angiogenesis and apoptosis of hepatic parenchymal cells, inhibiting activation of HSCs	Siliang Wang et al. (2021)

## TCMs treating hepatic fibrosis

**FIGURE 3:**
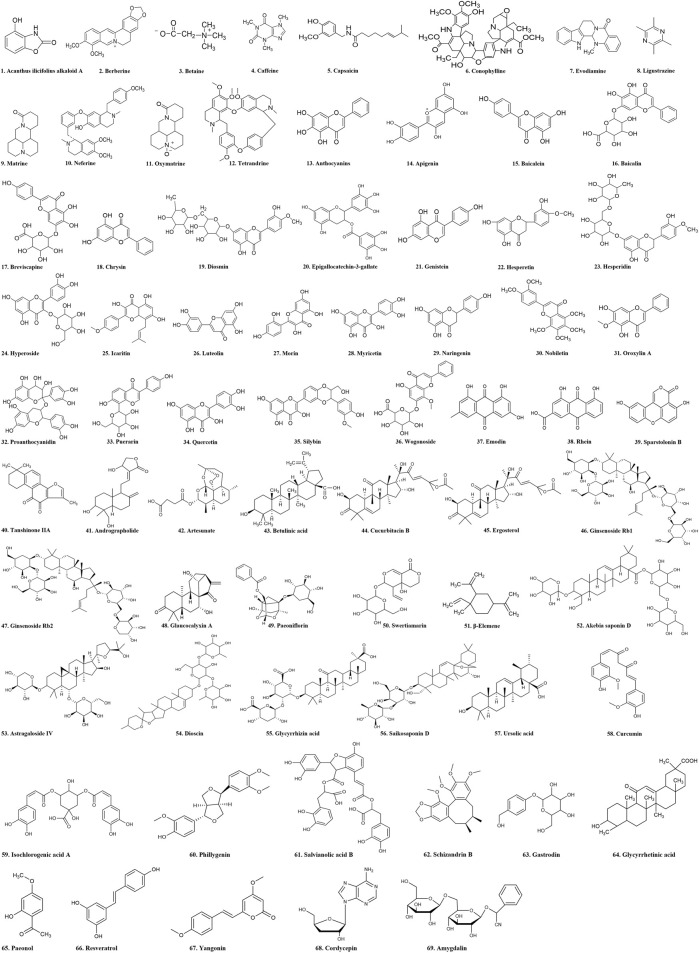
Chemical structural formula of natural products.

Nowadays, HF becomes a major public health problem with severe consequence (Berumen et al., 2021). Finding and discovering antifibrotic drugs from TCMs has become a hot research field. TCMs have unique advantages and great application prospects in anti-hepatic fibrosis due to their diverse structures, low toxicity, and wide sources (Daniyal et al., 2019; Hamza et al., 2020). Studies have shown that natural products derived from TCMs, such as alkaloids, flavones, quinones, terpenoids, saponins, phenylpropanols and polysaccharides, have promising anti-fibrotic activities (Shan et al., 2019). In addition, some TCMs and their formulas would be also beneficial for treating HF. The anti-fibrotic effects of these natural products were summarized in the following section according to the classification of chemical structure (Figure 3).

## Alkaloids

Alkaloids is a class of basic organic compounds containing nitrogen that mainly exist in plants or herbs. Most of them have complex ring structures, conjugation, and optical rotation. They have alkali like properties and significant biological activities, which are an important class of effective components in TCMs (Mondal et al., 2019). For example, acanthus ilicifolius alkaloid A, berberine and betaine are effective antioxidants with strong free radical scavenging effects, which can reduce the bio-markers’ levels of HF, so significantly improve liver functions (Wai et al., 2015; Eissa et al., 2018; Shedid et al., 2019). In addition, caffeine, capsaicin, evodiamine, matrine, neferine, oxymatrix and tetrandrine exert anti-fibrotic effects through regulating the TGF-β signal transduction pathway (Hsu et al., 2007; Gressner et al., 2008; Yu et al., 2014; Chen M. S. et al., 2015; Zhao et al., 2016; Choi et al., 2017; Yang et al., 2018). Besides, conophylline and ligustrazine can inhibit the activation of HSCs, and reduce the deposition of ECM (Kubo et al., 2014; Zhang et al., 2018). Table 2 summarized the anti-fibrotic effects of alkaloids found in TCMs against HF.

**TABLE 2:** Alkaloids with anti-hepatic fibrosis effect.

No.	Compounds	Source	Molecular formula	Mechanisms	Refs
**1**	Acanthus ilicifolius alkaloid A	*Acanthus ilicifolius* L.	C_7_H_5_NO_3_	Inhibiting inflammatory response;	Wai et al. (2015)
				Reducing lipid peroxidation and oxidative stress	
**2**	Berberine	*Coptis chinensis* Franch.	C_20_H_18_NO_4_	Antioxidant and anti-inflammatory responses	Eissa et al. (2018)
**3**	Betaine	*Beta vulgaris* L.	C_5_H_11_NO_2_	Reducing oxidative stress response	Shedid et al. (2019)
**4**	Caffeine	*Coffea arabica* L.	C_8_H_10_N_4_O_2_	Inhibiting TGF-β-induced CTGF expression by inhibiting Smad2/3 phosphorylation and up-regulating PPARγ receptor	Gressner et al. (2008)
**5**	Capsaicin	*Capsicum annuum* L.	C_18_H_27_NO_3_	Inhibiting TGF-β1/Smad pathway	Choi et al. (2017)
**6**	Conophylline	*Catharanthus roseusvar. albus Lochnera rosea* var. flava	C_44_H_50_N_4_O_10_	Inhibiting HSCs activation and induction of apoptosis	Kubo et al. (2014)
**7**	Evodiamine	*Euodia rutaecarpa* (Juss.) Benth	C_19_H_17_N_3_O	Inhibiting TGF-β1 and p-Smad2/3 signaling pathway expression	Yang et al. (2018)
**8**	Ligustrazine	*Ligusticum chuanxiong* hort.	C_8_H_12_N_2_	Inhibiting HSCs activation by interfering with PDGF-βR	Zhang et al. (2018)
**9**	Matrine	*Sophora flavescens* Alt.	C_15_H_24_N_2_O	Inhibiting TGF-β1 expression, enhance the activity of hepatocyte growth factor	Yu et al. (2014)
**10**	Neferine	*Nelumbo nucifera* Gaertn	C_38_H_44_N_2_O_6_	Decreasing expression of TGF-β1 in the liver	Mo-Si Chen et al. (2015)
**11**	Oxymatrine	*Sophora flavescens* Alt.	C_15_H_24_N_2_O_2_	Regulating TLR4-dependent inflammation and TGF-β1 signaling pathway	Zhao et al. (2016)
**12**	Tetrandrine	*Stephania tetrandra* S. Moore	C_38_H_42_N_2_O_6_	Inhibiting NF-κB transcriptional activity induced by TNF-α; Inhibiting TGF-β1-induced α-SMA secretion and collagen deposition in HSC-T6 cells	Hsu et al. (2007)

## Flavonoids

Flavonoids is a class of compounds with 2-phenyl chromogenic ketone structure as the basic parent core, which widely exists in berries and vegetables in nature. Flavonoids have notable antioxidant, anti-inflammatory, and anticancer activities, and can be used to treat a variety of liver diseases (Akhlaghi, 2016). For example, anthocyanins can significantly improve the severity of liver injury and fibrosis by reducing oxidative stress and inflammation (Sun et al., 2018). Epigallocatechin-3-gallate has anti-hepatic fibrosis effect *in vitro* by inhibiting PI3K/Akt/Smad pathway (Yu et al., 2015). Breviscapine, nobiletin, proanthocyanidin, silybin and wogonoside have anti-inflammatory and antioxidant activities, and these bioactive flavonoids can be used as promising agents for the treatment of HF and related liver diseases (Trappoliere et al., 2009; Li et al., 2012; Wang Q. et al., 2015; Liu Y. et al., 2018; Wu et al., 2020). Previous investigations showed that apigenin could improve CCl_4_-induced HF by mediating VEGF-mediated FAK phosphorylation through multiple pathways (Qiao et al., 2020). Baicalein, chrysin, icaritin and myricetin can prevent or reverse the progression of HF by inhibiting the activation of HSCs and inducing apoptosis of activated HSCs (Sun et al., 2010; Li et al., 2011; Balta et al., 2015; Geng et al., 2017). Besides, baicalin and diosmin exert anti-fibrotic effects by stimulating the expression of PPAR-γ (Qiao et al., 2011; Hasan et al., 2017). Genistein, hesperetin and luteolin can block the development of HF by decreasing TGF-β and activating TGF-β/Smad signaling (Li et al., 2015; Ganai and Husain, 2017; Kong et al., 2018). Moreover, hesperidin, naringenin, puerarin and quercetin inhibit liver tissue injury, necrosis, and fibrosis by reducing the activity of cytokines and biological receptors such as NF-κB (Pérez-Vargas et al., 2014; Wang et al., 2016; Wang R. et al., 2017; Hernández-Aquino et al., 2017). In addition, hyperoside and morin improve liver function and reduce the occurrence of HF by enhancing the expression of liver Nrf2 (Sang et al., 2017; Zou et al., 2017). Furthermore, oroxylin A can dose-dependently reduce biomarkers of liver injury and HSC activation by activating autophagy (Chen et al., 2018). Table 3 summarized the anti-fibrotic effects of flavonoids from TCMs against HF.

**TABLE 3:** Flavonoids with anti-hepatic fibrosis effect.

No.	Compounds	Source	Molecular formula	Mechanisms	Refs
13	Anthocyanins	*Vaccinium corymbosum* L.	C_15_H_11_O_6_	Regulating oxidative stress, inflammation and activation of HSCs	Sun et al. (2018)
**14**	Apigenin	*Apium graveolens* L.	C_15_H_10_O_5_	VEGF-mediated FAK phosphorylation through multiple pathways	Qiao et al. (2020)
**15**	Baicalein	*Scutellaria baicalensis Georgi*	C_15_H_10_O_5_	Downregulating PDGF-β receptor; inhibiting the activation and proliferation of HSC	Sun et al. (2010)
**16**	Baicalin	*Scutellaria baicalensis Georgi*	C_21_H_18_O_11_	Activating PPARγ and inhibiting TGF-β1	Qiao et al. (2011)
**17**	Breviscapine	*Erigeron breviscapus* (Vant.) Hand. -Mazz.	C_21_H_18_O_12_	Inhibiting inflammation and apoptosis	Yu Liu et al. (2018), Lan et al. (2022)
**18**	Chrysin	*Oroxylum indicum* (Linn.) Kurz	C_15_H_10_O_4_	Inhibiting HSCs activation and proliferation through TGF-β1/Smad pathway	Balta et al. (2015)
**19**	Diosmin	*Citrus aurantium* L.	C_28_H_32_O_15_	Boosting PPAR-γ expression and hampering miR-17-5p-activated canonical Wnt-β-catenin signaling	Hasan et al. (2017)
**20**	Epigallocatechin-3-gallate	*Acacia catechu* (L.f.) Willci.	C_22_H_18_O_11_	Inhibiting PI3K/Akt/Smad pathway	Yu et al. (2015)
**21**	Genistein	*Genista tinctoria* Linn.	C_15_H_10_O_5_	Blocking the TGF-β/Smad signaling pathway	Ganai and Husain, (2017)
**22**	Hesperetin	*Citrus reticulata* L.	C_16_H_14_O_6_	Inhibiting TGF-β1/Smad pathway	Kong et al. (2018)
**23**	Hesperidin	*Citrus reticulata* L.	C_28_H_34_O_15_	Decreasing the expression of NF-κB, TGF-β and connective tissue growth factor	Pérez-Vargas et al. (2014)
**24**	Hyperoside	*Hypericum perforatum* L.	C_21_H_20_O_12_	Activating Nrf2 nuclear translocation	Zou et al. (2017)
**25**	Icaritin	*Epimedium brevicornu* Maxim.	C_21_H_20_O_6_	Inducing activated HSCs death	Li et al. (2011)
**26**	Luteolin	*Reseda odorata* L.	C_15_H_10_O_6_	Inhibiting AKT/mTOR/p70S6K and TGF-β/Smad signaling pathway	Li et al. (2015)
**27**	Morin	*Cudrania cochin chinensis* (Lour.) Kudo et Masam.	C_15_H_10_O_7_	Enhancing hepatic Nrf2 expression	Sang et al. (2017)
**28**	Myricetin	*Myrica rubra* (Lour.) S. et Zucc.	C_15_H_10_O_8_	Inhibiting HSCs activation	Geng et al. (2017)
**29**	Naringenin	*Anacardium occidentalie* Linn.	C_15_H_12_O_5_	Inhibiting NF-κB, TGF-β-Smad3 and JNK-Smad3 signaling pathways	Hernández-Aquino et al. (2017)
**30**	Nobiletin	*Citrus nobilis* Lour.	C_21_H_22_O_8_	Reducing inflammation	Hernández-Aquino et al. (2017)
**31**	Oroxylin A	*Oroxylum indicum* (Linn.) Kurz.	C_16_H_12_O_5_	Activating autophagy	Chen et al. (2018)
**32**	Proanthocyanidin	*Vitis vinifera L.*	C_30_H_26_O_13_	Anti-lipid peroxidation	Li et al. (2012)
**33**	Puerarin	*Pueraria montanavar. lobata* (Willd.) Sanjappa & Pradeep	C_21_H_20_O_10_	Inhibiting PARP-1 and subsequent attenuating NF-κB, ROS production and mitochondrial dysfunction	Wang et al. (2016)
**34**	Quercetin	*Sophora flavescens* Ait.	C_15_H_10_O_7_	Reducing inflammation; Regulating NF-κB and p38 MAPK pathway	Rong Wang et al. (2017)
**35**	Silybin	*Silybum marianum* (L.) Gaertn.	C_25_H_22_O_10_	Anti-inflammatory and antioxidant activity	Trappoliere et al. (2009)
**36**	Wogonoside	*Scutellaria baicalensis* Georgi.	C_22_H_20_O_11_	Reducing inflammation	Qichao Wang et al. (2015)

## Quinones

Quinones is a kind of chemical constituents with quinone structure, which are mainly divided into four types: benzoquinone, naphthoquinone, phenanthrenequinone and anthraquinone. Quinones are widely distributed in plants and have various biological activities. Studies have shown that quinones have anti-inflammatory, anticancer, anti-viral and hepatoprotective activities, which lay a foundation for the treatment of HF (Dong X. et al., 2020). For example, rhein can significantly reduce alanine aminotransferase (ALT) activity in liver tissue of CCl_4_/ethanol-injured rats, improve the histological changes of HF, and decrease the expression of α-SMA and TGF-β1 in liver tissue. Therefore, rhein has a protective effect on CCl_4_/ethanol-induced liver injury in rats, inhibiting HF. Its mechanism of action may be related to antioxidant and anti-inflammatory effects, but can also inhibit TGF-β1 to stop HSC activation (Guo et al., 2002). Emodin is an active ingredient isolated from rhubarb, which has antimicrobial, immunosuppressive and anti-inflammatory effects. CCl_4_ was used to establish an experimental HF model in rats, and emodin was administered for treatment. The results suggested that Emodin might play an anti-fibrotic role by inhibiting TGF-β1 signal transduction to prevent HSC activation (Dong et al., 2009; Liu F. et al., 2018). Studies have found that tanshinone IIA can significantly improve liver function, alleviate liver injury, reduce ECM accumulation, inhibit HSC proliferation and activation, thereby playing an anti-fibrotic role. Tanshinone IIA may alleviate HF through multi-target and multi-signal pathways (Liu and Huang, 2014; Shi et al., 2020). In addition, sparstolonin B can play an anti-hepatic fibrosis role by inhibiting the TLR4 signal transduction pathway (Dattaroy et al., 2018). Table 4 summarized the anti-fibrotic effects of quinones from TCMs against HF.

**TABLE 4:** Quinones with anti-hepatic fibrosis effect.

No.	Compounds	Source	Molecular formula	Mechanisms	Refs
**37**	Emodin	*Rheum palmatum* L.	C_15_H_10_O_5_	Inhibiting HSC activation by mediating TGF-β/Smads signaling pathway	Dong et al. (2009), Feng Liu et al. (2018)
**38**	Rhein	*Rheum palmatum* L.	C_15_H_8_O_6_	Antioxidant and anti-inflammatory responses.	Guo et al. (2002)
**39**	Sparstolonin B	*Sparganium stoloniferum* (Graebn.), Buch-Ham. ex Juz.	C_15_H_8_O_5_	Inhibiting TLR4 signaling transduction pathway	Dattaroy et al. (2018)
**40**	Tanshinone IIA	*Salvia miltiorrhiza* Bge.	C_19_H_18_O_3_	Inhibiting HSC activation and proliferation	Liu and Huang, (2014), Stanković et al. (2017)

## Terpenoids

Terpenoids and their derivatives are the compounds with isoprene units as their basic structures widely existed in nature. Terpenoids have antioxidant, metabolic, immunomodulatory, and anti-inflammatory activities (Sánchez-Crisóstomo et al., 2019), and have promising potentials for the treatment of some hepatic chronic diseases. For example, cucurbitacin B attenuates HF by inhibiting oxidative stress, inflammation, and STAT3 signaling pathways (Sallam et al., 2018). β-Elemene could reduce plasma endotoxin level, serum tumor necrosis factor-alpha level and CD14 mRNA expression in liver of rats with HF rats by CCl_4_ treatment (Liu et al., 2011). In addition, andrographolide, ginsenoside Rb1 and paeoniflorin could inhibit HF by regulating the TGF-β1/Smads signaling pathway (Lo et al., 2011; Hu Z. et al., 2018; Lin et al., 2018), and artesunate, ergosterol, ginsenoside Rb2, glaucocalyxin A and swertiamarin can exert anti-fibrotic effects by inhibiting the activation of HSCs (Park et al., 2006; Tai et al., 2016a; Li S. et al., 2016; Dong et al., 2018; Kong et al., 2019). Betulinic acid exerts significant anti-fibrotic effects on rats treated with intraperitoneal injection of thioacetamide (TAA) by regulating TLR4/MyD88/NF-κB signaling pathway (Wan et al., 2012). Table 5 summarized the anti-fibrotic effects of terpenoids found in TCMs against HF.

**TABLE 5:** Terpenoids with anti-hepatic fibrosis effects.

No.	Compounds	Source	Molecular formula	Mechanisms	Refs
**41**	Andrographolide	*Andrographis paniculate* (Burm. f.) Nees	C_20_H_30_O_5_	Inhibiting the TGF-β1/Smad2 and TLR4/NF-κB p50 pathways	Lin et al. (2018)
**42**	Artesunate	*Artemisia annua* Linn.	C_19_H_28_O_8_	Inhibiting activation and proliferation of HSC; Inhibiting LPS/TLR4/NF-κB signaling pathway	Kong et al. (2019)
**43**	Betulinic acid	*Betula platyphylla* Suk.	C_30_H_48_O_3_	Inhibiting NF-κB signaling pathway	Wan et al. (2012)
**44**	Cucurbitacin B	*Cucumis melo* L.	C_32_H_46_O_8_	Inhibiting oxidative stress, inflammation and STAT3 signaling	Sallam et al. (2018)
**45**	Ergosterol	*Ganoderma lucidum* (Leyss. ex Fr.) Karst	C_28_H_44_O	Inhibiting HSCs activation	Tai et al. (2016a)
**46**	Ginsenoside Rb1	*Panax ginseng* C.A. Mey	C_54_H_92_O_23_	Regulating the expression of collagen, TGF-β1, MMP-2 and TIMP-1	Lo et al. (2011)
**47**	Ginsenoside Rb2	*Panax ginseng* C.A. Mey	C_53_H_90_O_22_	Inducing apoptosis in HSC via caspase-3 activation	Park et al. (2006)
**48**	Glaucocalyxin A	*Rabdosia japonica* (Burm.f.) Hara var.glaucocalyx (Maxim.) Hara	C_20_H_28_O_4_	Attenuating the activation of HSC through the TGF-β1/Smad signaling pathway	Dong et al. (2018)
**49**	Paeoniflorin	*Paeonia lactiflora* Pall.	C_23_H_28_O_11_	Inhibiting TGF-β1/Smads signaling pathway	Zongtao Hu et al. (2018)
**50**	Swertiamarin	*Gentianaceae Swertia* L.	C_16_H_22_O_10_	Inhibiting HSC activation by regulating the RAS	Shu Li et al. (2016)
**51**	β-Elemene	*Curcuma Wenyujin* Y.H.Chen et C.Ling	C_15_H_24_	Regulating endotoxin signaling transduction pathway	Liu et al. (2011)

## Saponins

Saponins, a class of glycosides whose aglycones are triterpenoids or spirosterols, are complex compounds in the structure, which widely exist in plants and have a wide variety of species. Saponins have antioxidant, anticancer and multi-target activities, which play an important regulatory role in the treatment of HF (Li X. et al., 2018). For example, akebia saponin D and saikosapoin D can reduce HF through anti-inflammatory effects (Dang et al., 2007; Gong et al., 2019), and astragaloside IV, glycyrrhizin acid and ursolic acid could alleviate HF by inhibiting HSC activation and inducing apoptosis of activated HSCs (Wang et al., 2011; Li et al., 2013; Liang et al., 2015). In addition, dioscin can specifically inhibit collagen synthesis by regulating the PI3K/Akt pathway, and can be used as an effective anti-fibrotic drug (Xu et al., 2017). Table 6 summarized the anti-fibrotic effects of saponins found in TCMs against HF.

**TABLE 6:** Saponins with anti-hepatic fibrosis effect.

No.	Compounds	Source	Molecular formula	Mechanisms	Refs
**52**	Akebia saponin D	*Dipsacus asperoides* C.Y. Cheng et T.M.Ai.	C_47_H_76_O_18_	Anti-inflammatory effect	Gong et al. (2019)
**53**	Astragaloside IV	*Astragalus membranaceus* (Fisch.) Bungede	C_41_H_68_O_14_	Inhibiting HSC activation by inhibiting oxidative stress and p38 MAPK signaling pathway	Li et al. (2013)
**54**	Dioscin	*Dioscorea japonica* Thunb.	C_45_H_72_O_16_	Inhibiting collagen synthesis by regulating PI3K/Akt pathway	Xu et al. (2017)
**55**	Glycyrrhizin acid	*Glycyrrhize glabra* L.	C_42_H_62_O_16_	Inhibiting hepatocyte apoptosis and activation of HSCs	Liang et al. (2015)
**56**	Saikosaponin D	*Bupleurum chinense* DC.	C_42_H_68_O_13_	Reducing inflammation and hepatocyte injury	Dang et al. (2007)
**57**	Ursolic acid	*Prunella vulgaris* L.	C_30_H_48_O_3_	Inducing apoptosis of activated HSCs	Wang et al. (2011)

## Phenylpropanoids

Phenylpropanoids is a kind of natural compounds composed of benzene ring and three straight chain carbon, and have anti-tumor, antioxidant and hepatoprotective effects (Teponno et al., 2016). For example, curcumin can significantly improve the severity of liver injury and fibrosis by reducing oxidative stress and inflammation (Kong et al., 2020). Experimental studies have found that the anti-fibrotic mechanism of salvianolic acid B may be through inhibiting HSC proliferation (Liu et al., 2002). In addition, isochlorogenic acid A could delay the progression of HF by regulating HMGB1/TLR4/NF-κB signaling pathway (Liu et al., 2020). Schizandrin B plays an anti-fibrotic role by regulating Nrf2-ARE and TGF-β/Smad signaling pathways (Chen et al., 2017). Phillygenin is a phenylpropanoid compound isolated from Forsythia and has good anti-inflammatory effects. Phillygenin inhibits LPS induced proinflammatory response and Lx2 cell activation through TLR4/MyD88/NF-κB signaling pathway, thereby inhibiting HF (Hu et al., 2020b). Table 7 summarized the anti-fibrotic effects of phenylpropanoids found in TCMs against HF.

**TABLE 7:** Phenylpropanoids with anti-hepatic fibrosis effect.

No.	Compounds	Source	Molecular formula	Mechanisms	Refs
**58**	Curcumin	*Curcuma longa* L.	C_21_H_20_O_6_	Regulating oxidative stress and autophagy, passivation of hepatocyte epithelial-mesenchymal transformation	Kong et al. (2020)
**59**	Isochlorogenic acid A	*Lonicera japonica* Thunb.	C_25_H_24_O_12_	Inhibiting HMGB1/TLR4/NF-κB signaling pathways	Liu et al. (2020)
**60**	Phillygenin	*Forsythia suspensa* (Thunb.) Vahl	C_21_H_24_O_6_	Regulating TLR4/MyD88/NF-κB signaling pathways	Hu et al. (2020b)
**61**	Salvianolic acid B	*Salvia miltiorrhiza* Bge.	C_36_H_30_O_16_	Inhibiting HSC proliferation and collagen synthesis, Decreasing TGF-β1 autocrine and MAPK activity	Liu et al. (2002)
**62**	Schizandrin B	*Schisandra chinensis* (turcz.) baill.	C_23_H_28_O_6_	Regulating Nrf2-ARE and TGF-β/Smad signaling pathways	Chen et al. (2017)

## Polysaccharides

Polysaccharides are polysaccharide macromolecular carbohydrates consisting of glycosidic bonded sugar chains with at least 10 monosaccharides. Polysaccharides are widely distributed in nature and have the characteristics of multi-component, multi-target, high efficiency, and low toxicity (Qu et al., 2020). In recent years, increasing investigations reported that polysaccharides possess promising hepatoprotective effects against HF. Table 8 summarized the anti-fibrotic effects of polysaccharides found in TCMs against HF.

**TABLE 8:** Polysaccharides with anti-hepatic fibrosis effect.

Polysaccharides	Source	Mechanisms	Refs
Astragalus polysaccharides	*Astragalus membranaceus* (Fisch.) Bge.	Inhibiting LX-2 cell proliferation	Zheng et al. (2014)
Cordyceps polysaccharides	*Cordyceps sinensis* (BerK.) Sacc.	Reducing ROS production	Liu et al. (2013)
Dicliptera chinensis polysaccharides	*Dicliptera chinensis* (L.) Juss.	Reducing inflammation	Kefeng Zhang et al. (2016)
Fucoidan	*Fucus vesiculosus*	Inhibiting TGF-β1/Smads signaling pathway	Jingjing Li et al. (2016)
Garlic polysaccharide	*Allium sativum* L.	Inhibiting intestinal flora imbalance	Yuchuan Wang et al. (2018)
Lycium barbarum polysaccharides	*Lyciumbarbarum* L.	Inhibiting TLRs/NF-κB signaling pathway expression	Gan et al. (2018)
Polysaccharide from Amusium Pleuronect	*Amusium Pleuronectes pleuronectes Linnaeus*	Hepatoprotective effect	Tang et al. (2018)

## Other natural compounds

Many other compounds extracted from TCMs have therapeutic effects on HF through complex mechanisms. Polyphenols have been reported to have good effects in alleviating oxidative stress, lipid metabolism and inflammation (Luk et al., 2007; Li S. et al., 2018). For example, gastrodin can significantly improve the severity of liver injury and fibrosis by reducing oxidative stress and inflammation (Zhao et al., 2014; Zhao et al., 2015). Glycyrrhetinic acid protects the liver by activating the nuclear translocation of Nrf2 and increasing the activity of antioxidant enzymes. Therefore, glycyrrhetinic acid may be an effective hepatoprotective drug for the treatment of HF (Chen et al., 2013; Lam et al., 2016). Experimental studies have found that the anti-fibrotic mechanism of paeonol may be through inhibiting HSC proliferation (Kong et al., 2013; Tai et al., 2016b). Resveratrol delays the progression of HF by reprogramming macrophage phenotype from LPS to IL-4 by upregulating endogenous IL-10 (Hsu et al., 2019; Yu et al., 2019). The anti-fibrotic mechanism of yangonin may be that it reduces collagen content by regulating HF-related genes Col1-α-1 and TIMP-1 (Cao et al., 2018; Wang X. et al., 2019). In addition, Lan et al. (2021) established the fibrosis model in mice induced by 16 weeks of high-fat/high-cholesterol diet administration. They found that cordycepin (100 and 200 mg/kg) treatment significantly inhibited the expression of fibrogenic genes such as ColIa1, ColIIIa1, α-SMA, and decreased the serum levels of ALT and aspartate aminotransferase (AST). It is revealed that cordycepin plays an important protective role in hepatic fibrosis mice. Furthermore, Wang R. et al. (2021) demonstrated the suppressive effects of amygdalin in TGF-β1-induced HSC activation *in vitro* and found that amygdalin (3 mg/kg) could reduce the levels of ALT and AST in serum, decrease the levels of α-SMA, collagen I, and TIMP-1 in hepatic tissue samples, improve focal necrosis and collagen fiber accumulation in CCl_4_-induced hepatic fibrosis rats. Table 9 summarized the anti-fibrotic effects of other compounds found in TCMs against HF.

**TABLE 9:** Other natural compounds with anti-hepatic fibrosis effect.

No.	Compounds	Source	Molecular formula	Mechanisms	Refs
**63**	Gastrodin	*Gastrodiaelataf glauca*	C_13_H_18_O_7_	Reducing oxidative stress and inflammation	Zhao et al. (2014), Qu et al. (2016)
**64**	Glycyrrhetinic acid	*Glycyrrhize glabra* L.	C_30_H_46_O_4_	Hepatoprotective effect	Lam et al. (2016)
**65**	Paeonol	*Paeonia moutan* Sim.	C_9_H_10_O_3_	Inhibiting HSC production and NF-κB pathway	Tai et al. (2016b)
**66**	Resveratrol	*Veratrum nigrum* L.	C_14_H_12_O_3_	Up-regulating endogenous IL-10 to reprogramme macrophages phenotype from LPS to IL-4	Hsu et al. (2019)
**67**	Yangonin	*Piper methysticum* Forst.	C_15_H_14_O_4_	Activating farnesoid X receptor	Cao et al. (2018)
**68**	Cordycepin	*Cordyceps sinensis* (Berk.) Sacc.	C_10_H_13_N_5_O_3_	Activating AMPK signaling pathway	Lan et al. (2021)
**69**	Amygdalin	*Prunus armeniaca* L.var.ansu Maxim	C_20_H_27_NO_11_	Inhibiting HSC proliferation and activation	Ruoyu Wang et al. (2021)

### Other extracts from TCMs

Besides, some TCM extracts are also beneficial for the treatment of HF via protecting hepatocytes and inhibiting hepatitis and fibrosis (Wang Y. et al., 2015; Wang Y. Y. et al., 2017; Miao et al., 2019). Table 10 summarized the anti-fibrotic effects of these extracts.

**TABLE 10:** Other extracts from TCM with anti-hepatic fibrosis effect.

Extracts	Source	Mechanisms	Refs
Water extracts from *Solanum nigrum*	*Solanum nigrum* L.	Downregulating HSCs activation by inhibiting advanced glycation end products-associated signaling pathways involved in anti-glycation and Nrf2 activity	Sha Li et al. (2018)
*Graptopetalum paraguayense*	*Graptopetalum paraguayense* E. Walther	Regulating HSC activation by the Inhibiting the TGF-β pathway	Zhao et al. (2015)
*Semen brassicae*	*Brassica campeatris* L.	Regulating TGF-β1/Smad, NF-κB, and AKT signaling pathways and the reduction of extracellular matrix deposition	Chen et al. (2013)
Water extract of *Lonicerae japonicae*	*Lonicera japonica* Thunb.	Inhibiting HSC activation, reducing hepatic ECM deposition, ameliorating liver oxidative stress injury and reversing EMT	Kong et al. (2013)
*Ginkgo biloba* extract	*Ginkgo biloba* L.	Regulating p38, MAPK and NF-κB/IκBα signaling to inhibit HSC activation and disrupting Bcl-2/Bax signaling to inhibit hepatocyte apoptosis	Yu et al. (2019)
*Glechoma hederacea* extracts	*Glechoma longituba* (Nakai) Kupr.	Downregulating NF-κB, AP-1 and TGF-β/Smad signals by interfering with HMGB1/TLR4 axis	Xiaohui Wang et al. (2019)
Germacrone	Zedoary turmeric oil	Inhibiting the activation and survival of HSCs	Li et al. (2021)
Total C-21 steroidal glycosides from *baishouwu*	*Roots of Cynanchum auriculatum* Royle ex Wight	Regulating IL-1β/MyD88 inflammation signaling	Qin et al. (2021)
Red raspberry extract	*Rubus idaeus* L.	Inducing apoptosis and trans differentiation of activated HSCs	Wu et al. (2021)

## Clinical trials

To date, there are several clinical trials on the anti-hepatic fibrosis effect of TCMs. Chronic hepatitis B (CHB) can progress to liver fibrosis, leading to cirrhosis with liver-related disease, and ultimately markedly increasing the risk of hepatocellular carcinoma (HCC) (Inoue and Tanaka, 2020). At present, a randomized, double-blind, and placebo-controlled clinical trial was carried out to estimate the antifibrotic activities of Anluo huaxian pill (AHP) on chronic hepatitis B (CHB) patients with advanced fibrosis or cirrhosis. The results found that the rate of histological improvement in patients with liver fibrosis was significantly higher in the AHP group than in the placebo group, the level of liver stiffness measurement (LSM) decreased dramatically from baseline in the AHP group but not in the placebo group, after orally administration with AHP at a dose of 6 g, twice daily for 48 weeks (Xiao et al., 2022), which indicated that AHP could improve liver fibrosis and may be useful as a functional food medicine. Besides, cirrhosis developed in one patient in the placebo group but in none of the patients in the AHP group, and no serious side effects occurred in patients treated with AHP (Xiao et al., 2022). Moreover, a multicenter, randomized, double-blind, placebo-controlled trial, patients with CHB were randomly assigned to receive entecavir (ETV) (0.5 mg/day)+FBRP (2 g three times/day), after 72 weeks of treatment, the rate of regression of fibrosis/cirrhosis was significantly higher in the ETV + BR group than ETV monotherapy in overall patients with CHB and those with liver cirrhosis at baseline, and this combination therapy also showed an excellent safety profile (Rong et al., 2022). To evaluate the efficacy and safety of Chunggan extract (CGX), Joung et al. (2020) conducted a randomized controlled clinical trial of CGX in patients with liver fibrosis diagnosed by Fibroscan. Orally administration with CGX (1 g or 2 g, twice daily) to the subjects for 24 weeks could dramatically decreased the LSM scores compared to the placebo group, and no notable adverse events were present, which firstly demonstrated that the potent pharmacological effects of CGX in restoring early liver fibrosis in patients with chronic liver disease. Based on a collective research report, luteolin, quercetin, ursolic acid and salvianic acid A are the main potential active components of FZHY formula to exert anti-inflammatory and anti-hepatic fibrosis pharmacological effects (Xin et al., 2022). Furthermore, the active components of FBRP to prevent HF mainly include total flavonoids from Astragalus membranaceus, salvianic acid A, notoginseng saponins, cordyceps polysaccharides (Yao et al., 2022). More notably, among these biological compounds from TCMs similarly positively affect fibrosis, as reported in the previous section.

At least, these established evidence indicated that TCMs might be considered as a viable, efficacious supplement for hepatic fibrosis, and can be used in daily life to prevent associated with liver disease. However, there is insufficient evidence to support the optimal dose of TCMs and what kind of hepatic fibrosis it has effect on deserve further research. Besides, the underlying mechanisms for the benefit of these TCMs therapy is unclear, and the specific medicinal components of TCMs responsible for the antifibrotic effects remain unknown. Therefore, it is necessary to investigate the effects of TCMs blockade and reversal of hepatic fibrosis in a larger, more rigorous methodological design and more diverse samples.

## Conclusion and prospect

**FIGURE 4:**
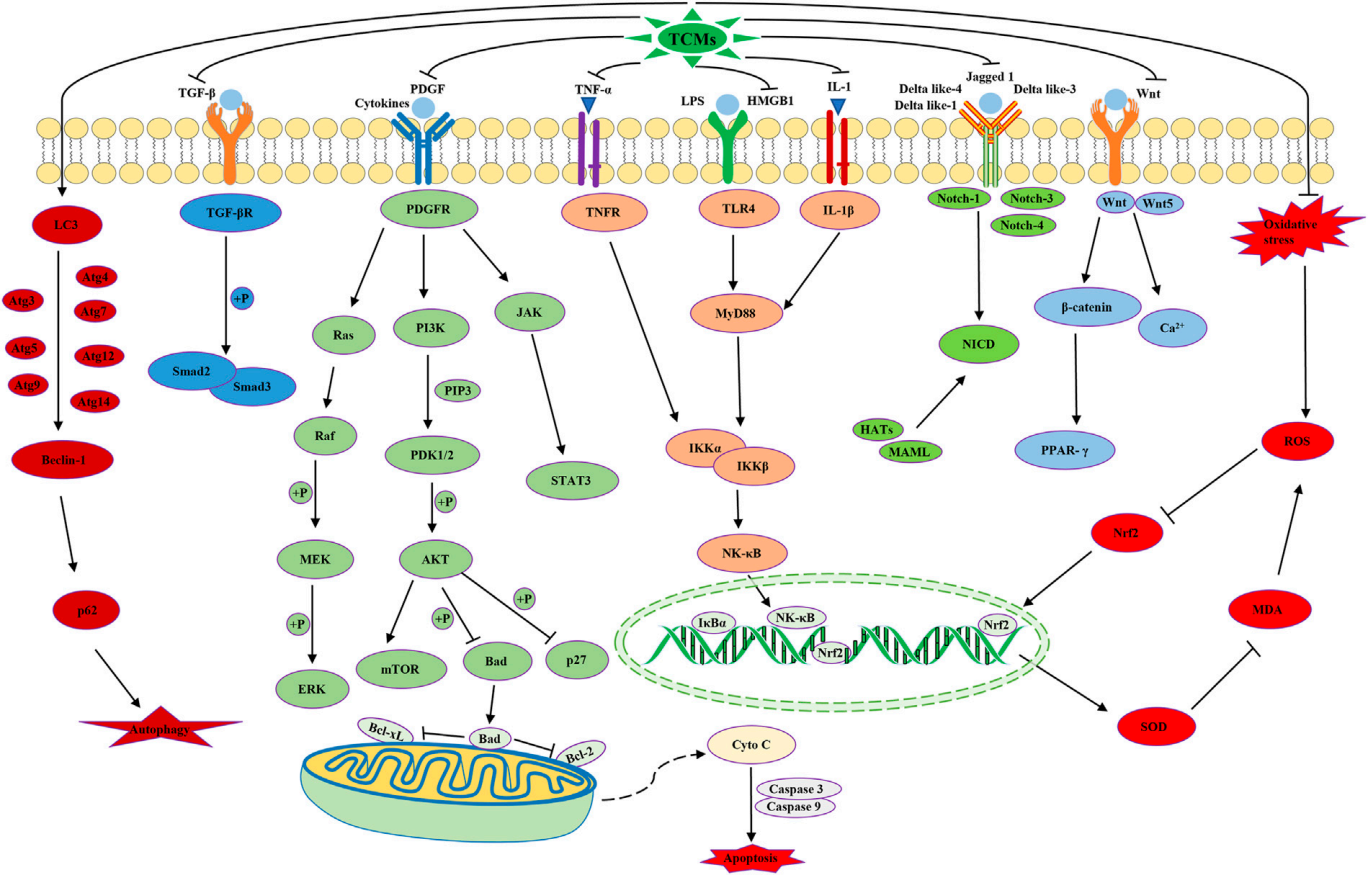
Schematic representation of the possible mechanism of TCMs on HF.

Hepatic fibrosis (HF) is a complex pathological process related to multiple factors, targets, and pathways, which causes serious harm to human health. In recent years, many TCMs and their active ingredients have been found to be effective in treating HF via multiple mechanisms (Figure 4). However, most of them remain at the stage of *in vitro* and *in vivo* experimental studies, which require more detailed preclinical and clinical studies.

First, clinical studies on anti-hepatic fibrosis ingredients derived from TCMs are lacking, the assessment of pre-clinical safety is rarely documented, and the possible side effects and toxicity are still uncovered. Therefore, more work should be devoted to clinical data mining and experimental research in the future. Second, there is currently insufficient evidence to explain the specific molecular mechanisms of these anti-fibrotic components. Therefore, the pharmacokinetics and pharmacological properties of these natural anti-hepatic fibrosis products from TCMs are needed to be further studied. Third, the current research on anti-hepatic fibrosis drugs mainly focuses on plant medicines, while the research on animal and mineral drugs is less. Therefore, future research on anti-hepatic fibrosis drugs can also involve more animal or mineral drugs.

In conclusion, TCMs have more advantages in the treatment of HF due to its unique theoretical system and long-term practice clinically. This review systematically summarized the potential anti-hepatic fibrosis components from TCMs and their related molecular mechanisms, which would be helpful for further investigations on TCMs derived remedies for HF. In addition, it is hoped that this review will inspire more scholars to make more contributions to the study of TCMs in the treatment of HF.
